# Identification of Dietary Patterns Associated with Incidence of Hyperglycemia in Middle-Aged and Older Korean Adults

**DOI:** 10.3390/nu11081801

**Published:** 2019-08-04

**Authors:** Kyung Won Lee, Hae Dong Woo, Mi Jin Cho, Jae Kyung Park, Sung Soo Kim

**Affiliations:** Division of Epidemiology and Health Index, Center for Genome Science, Korea National Institute of Health, Korea Centers for Disease Control and Prevention, Chungcheongbuk-do 28160, Korea

**Keywords:** dietary pattern, factor analysis, type 2 diabetes, hyperglycemia, prospective study, Korean adults, KoGES

## Abstract

Little is known about the association between dietary patterns and hyperglycemia incidence among Korean adults. Hence, we aimed to prospectively investigate the major dietary patterns associated with hyperglycemia among middle-aged and older Korean adults. In total, 55,457 adults (18,292 men and 37,165 women) aged 40 to 79 years, who were previously enrolled in the Health Examinee Study of the Korean Genome and Epidemiology Study and had no history of type 2 diabetes mellitus (T2DM) or cancer at baseline, were included. Dietary patterns were identified by a factor analysis based on dietary data, which were assessed at baseline using a validated food-frequency questionnaire. Participants were classified as having hyperglycemia if fasting blood glucose levels were ≥126 mg/dL or physician diagnosed T2DM during follow-up. Multivariable Cox proportional hazard models were used to examine the associations between each dietary pattern and future hyperglycemia risk after adjusting for potential confounders. After a mean follow-up of 4.9 years, 2574 new cases of hyperglycemia were identified. Using a factor analysis, four distinct dietary patterns were identified: “prudent;” “fatty fish, meat, and flour-based food;” “coffee and sweets;” and “whole grain (men)” or “white rice (women).” The “prudent” pattern was inversely associated with hyperglycemia risk only in women (hazard ratio [HR], 0.75; 95% confidence interval [CI], 0.63–0.89; *p* for trend = 0.0003). Conversely, women in the highest quintile of the “fatty fish, meat, and flour-based food” pattern showed an increased risk of hyperglycemia (HR, 1.22; 95% CI, 1.03–1.44; *p* for trend = 0.0210) compared with those in the lowest quintile. The “coffee and sweets” and “white rice” patterns were not associated with hyperglycemia risk in women. The dietary patterns observed in men had no associations with hyperglycemia incidence. Our findings suggest that a diet rich in vegetables, mushrooms, seaweeds, fruits, and soy products and low in fatty fish and high-fat meat may potentially play a protective role in T2DM development with sex differences in middle-aged and older Korean adults.

## 1. Introduction

Type 2 diabetes mellitus (T2DM) is one of the most prevalent chronic diseases in Korea. According to the 2016 Korea National Health and Nutrition Examination Survey, the prevalence of T2DM reached 13% in Korean adults aged 30 years and older, and the prevalence has been increasing in both men and women for the last 10 years [1]. The age-specific prevalence of T2DM increased with age and reached the peak (27.3%) in adults aged ≥65 years [1]. Since the population across Korea is rapidly aging, the increasing economic burden from diabetes has become a high priority in public health in the country [2].

T2DM is a multifactorial disease; various factors such as diet, physical activity, lifestyle, and genetics are involved in its development [3]; in particular, a growing body of evidence indicated the importance of diet in the development of T2DM [4]. Many experimental and observational studies proposed that some foods and nutrients have protective or adverse effects on the T2DM risk [5,6]. However, people consume a variety of foods together rather than separately, and the nutrients in these foods interact with each other. Studying one food or nutrient cannot capture the synergistic or antagonistic effects of the interactions between nutrients. The dietary-patterning analysis, which has attracted considerable interest, is an alternative approach that may overcome the limitation of a single food or nutrient approach [7].

Some epidemiological studies conducted in Korean populations have pointed out that empirically-derived dietary patterns could be associated with blood glucose levels, although the results were not always consistent. Findings from previous studies conducted in Korean adults indicated that those who had a certain dietary pattern, characterized by a high intake of fruit and dairy products and a low intake of refined grains had a reduced risk of impaired fasting glucose [8,9]. Using the data from the Korea National Health and Nutrition Examination Survey, Shin et al. [10] identified two dietary patterns (the meat/fast food pattern and the traditional dietary pattern), but neither was associated with a prevalence of elevated blood glucose levels. However, previous investigations focused on the prediabetic stage as a component of the metabolic syndrome rather than diabetes. Moreover, even though literature have mentioned the differences in eating habits, food choices, and diet–disease associations between men and women [11,12], previous studies did not consider the difference in dietary patterns by sex. Little is known about the associations between dietary patterns and hyperglycemia incidence and whether these associations vary by sex. Therefore, prospective studies exploring major dietary patterns that can contribute to the development of hyperglycemia in both sexes are warranted. Thus, the present study aimed to identify the distinct dietary patterns and sex-specific associations with the future risk of developing hyperglycemia in middle-aged and older Korean adults.

## 2. Materials and Methods

### 2.1. Data Source and Study Population

**Figure 1:**
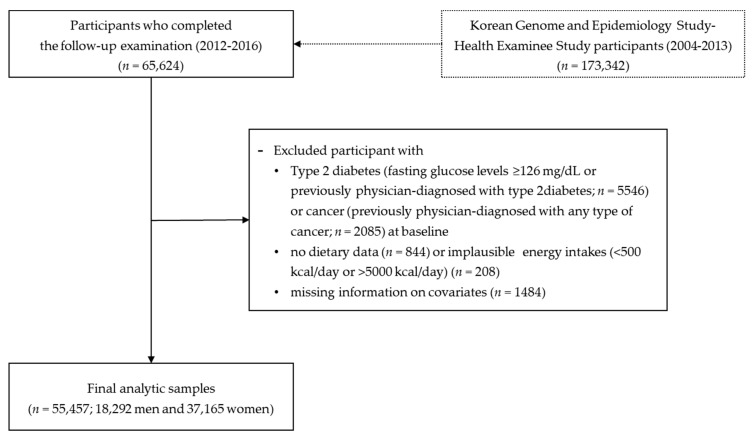
Flowchart of study population selection.

We used the data from the Korean Genome and Epidemiology Study (KoGES), which is a large prospective cohort project that investigated the environmental and genetic factors affecting prevalent chronic diseases in the Korean population [13]. As part of the KoGES, the population-based Health Examinee (HEXA) study recruited 173,342 Korean men and women (40–79 years of age) who had visited hospitals and public health centers across the country for biennial health check-ups. Between 2004 and 2013, the baseline data of each participant (socio-demographic characteristics, past medical history, family history, dietary behaviors, and reproductive health (for women)) were obtained by interviewing participants using structured questionnaires. Health examination and blood and urine tests were carried out by trained examiners using standardized procedures and protocols. The current study used data from 65,624 participants, who completed the follow-up survey between 2012 and 2016 (Figure 1). Of them, participants with T2DM (*n* = 5546) or cancer (any type) (*n* = 2085) at baseline were excluded. We also excluded individuals who had no dietary data (*n* = 844) or reported implausible dietary intake (<500 kcal/day or >5000 kcal/day) (*n* = 208) and those who had missing information on covariates (*n* = 1484). After the above exclusions, 55,457 Korean adults (18,292 men and 37,165 women) were included in the final analyses.

### 2.2. Dietary Assessment

The usual dietary intake of the study participants was assessed using a 106-item semi-quantitative food-frequency questionnaire (FFQ), which was specially designed to assess dietary intakes of KoGES participants. The validity and reproducibility of the FFQ has been previously evaluated against four 3-day dietary records [14]. Interviewers asked the participants how often they had consumed each food and beverage item on the list during the previous year. Nine possible frequency responses were provided, ranging from “never/seldom” to “three or more times a day.” Three of the nine responses were related to the portion size of each food item: “small” (one half the standard portion size), “medium” (one standard portion size), and “large” (two standard portion sizes). The usual intake of 106 food and beverage items was converted to a daily intake based on the participants’ responses about consumption frequencies and amounts. The daily total energy and nutrient intakes were calculated from the CAN-Pro 2.0 nutrient database, developed by the Korean Nutrition Society. All food and beverage items consumed by participants were aggregated into 37 food groups according to the grouping schemes commonly used in the Korean nutrient database [15] and the previous studies [10,16,17] (Table 1).

### 2.3. Outcome Variable

The primary endpoint of this study was the incidence of hyperglycemia. New hyperglycemia cases were identified on the basis of fasting blood glucose levels and self-reported physician diagnosis. Participants who had fasting blood glucose levels of 126 mg/dL or higher in the follow-up examination or who had been diagnosed with T2DM during the follow-up period were categorized as incident cases. Participants who had been free of hyperglycemia at the date of the last follow-up were censored at that time. Person-years were calculated from the date of baseline examination until the date of last follow-up or the date of the examination with new-onset of hyperglycemia, whichever occurred first.

### 2.4. Statistical Analyses

Major dietary patterns were identified for men and women separately to consider sex differences in dietary effects on hyperglycemia pathology. We conducted a factor analysis based on the total amount of each food and beverage group using the principal component analysis method (FACTOR procedure in SAS). To achieve a simpler structure and better interpretability, factor rotation (varimax) was applied. Four dietary patterns (factors) were retained based on the eigenvalue (≥1.6), scree test results, and interpretability of each factor for men and women, separately. Food groups with factor loading values ≥|0.30| were defined as important contributors to each dietary pattern and were used to characterize each pattern. The median and range values of factor scores by each dietary pattern quintile are listed in Supplementary Table S1. Major dietary patterns derived by factor analysis were relatively identical between men and women, except for the fourth pattern (“whole grain” pattern in men and “white rice” pattern in women). The factor score for each dietary pattern was calculated by summing the intake of each food group and multiplying that number by the corresponding factor loadings. A higher factor score indicated a higher level of adherence to a certain dietary pattern. Study participants were classified into quintiles according to the factor score of each dietary pattern for further analyses.

The baseline characteristics and nutrient intake of participants across the quintiles of dietary patterns were assessed using the multiple linear regression for continuous variables and the Mantel–Haenszel chi-square test for categorical variables. Continuous variables were expressed as means ± standard deviations, while categorical variables were expressed as frequencies (percentages). We used multivariable Cox proportional hazard models to estimate hazard ratios and 95% confidence intervals, with the lowest quintile as the reference. The following covariates were included: age (years), education (≤elementary school, middle school, high school, or ≥college), smoking habits (never, past, or current), total alcohol intake (g/day), regular physical activity (yes or no), family history of diabetes (yes or no), study site, body mass index (kg/m^2^), fasting blood glucose level (mg/dL), and total energy intake (kcal/day) at the baseline examination. Linear trends across the quintiles of each dietary pattern were tested. All statistical analyses were performed using the SAS software (version 9.4; SAS Institute, Inc., Cary, NC, USA). A two-sided *p*-value of less than 0.05 was considered significant.

## 3. Results

The factor analysis identified four major dietary patterns according to sex. The factor loadings for each dietary pattern are shown in Table 1. The pattern labelled as “prudent” was characterized by high consumption of vegetables, mushrooms, seaweeds, fruits, soy products, and milk. The “fatty fish, meat, and flour-based food” pattern was characterized by high intake of fatty fish, high-fat red meat, poultry, bread, noodles/dumplings, pizza/hamburger, and carbonated beverages. The “coffee and sweets” pattern was characterized by high intakes of coffee, sweets, and oils/fats. Lastly, the “whole grain” pattern, which was characterized by extremely high consumption of whole grain, was identified in men, and the “white rice” pattern, which was characterized by a very high intake of white rice with a very low intake of whole grain, was observed in women.

The baseline characteristics of study participants across quintiles of the dietary pattern scores are described in Table 2 and Table 3. Participants adhering to the “prudent” pattern were more likely to be older in age and have a higher education level. They also tended to maintain healthy behaviors, such as doing regular exercise. On the contrary, individuals with a higher factor score for the “fatty fish, meat, and flour-based food” pattern were younger, tended to smoke more, consumed more alcohol, and exercised less regularly. Participants adhering the “coffee and sweets” pattern were younger and less physically active, and tended to be current smokers both in men and women. Men in the highest quintile of the “whole grain” pattern were more likely to be younger and have higher levels of education and physical activity, but were less likely to be current smokers and consume alcohols. Women who had high scores for the “white rice” pattern were more likely to be current smokers and drink alcohol, and less likely to have a higher education level and perform regular exercise.

Nutrient intakes across the quintiles of each dietary pattern score are shown in Table 4 and Table 5. Total energy intake increased with higher factor scores for all dietary patterns, excluding the “white rice” pattern, derived in this study. However, we found there were differences in trends of energy and nutrient intakes among the various dietary patterns. In both men and women, individuals in the highest quintile of the “prudent” pattern acquired less energy from carbohydrates but more energy from protein and fat, and had higher intake of all other nutrients compared with those in the lowest quintile. On the contrary, men and women with the higher “fatty fish, meat, and flour-based food” pattern scores showed negative associations with intake of most nutrients, except for protein and fat. Individuals in the highest quintile of the “coffee and sweets” pattern had lower intakes of carbohydrates, protein, phosphorus, iron, vitamin A, carotene, folate, and dietary fiber, but they had higher intakes of fat, sodium, and potassium regardless of sex. Men having the “whole grain” pattern showed higher intakes of calcium, phosphorus, iron, vitamin C, folate, and dietary fiber, but lower intakes of protein, sodium, vitamin A, and carotene.

During the average follow-up of 4.9 years, we identified 2574 (4.6%) new cases of hyperglycemia. Table 6 shows the associations of major dietary patterns with the risk of hyperglycemia development. The “prudent” and “fatty fish, meat, and flour-based food” patterns were significantly associated with hyperglycemia incidence in women. Compared with women in the lowest quintile of the “prudent” pattern, those in the highest quintile were less likely to develop hyperglycemia (hazard ratio [HR], 0.75; 95% confidence interval [CI], 0.63–0.89; *p* for trend = 0.0003) after adjusting for age, education, smoking status, alcohol intake, physical activity, family history of diabetes, study site, body mass index, fasting blood glucose levels, and total energy intake at baseline. In the age-adjusted model, women who had the “fatty fish, meat, and flour-based food” pattern had a higher incidence of hyperglycemia (HR for fifth vs. first quintile, 1.39; 95% CI, 1.17–1.65; *p* for trend = 0.0002) than those who did not adhere this dietary pattern. These associations became attenuated but remained significant when all covariates were included in the model (HR for fifth vs. first quintile, 1.22; 95% CI, 1.03–1.44; *p* for trend = 0.0210). Other two dietary patterns, the “coffee and sweets” and “white rice” patterns, showed no significant associations with a risk of developing hyperglycemia for women. None of the dietary patterns identified in men were associated with an increased or decreased risk of hyperglycemia development.

## 4. Discussion

In this large-scale, population-based cohort study, we investigated the major dietary patterns and their prospective associations with hyperglycemia risk among middle-aged and older Korean adults. Based on the results of our factor analysis, we identified three similar dietary patterns for both Korean men and women: “prudent;” “fatty fish, meat, and flour-based food;” and “coffee and sweets” patterns. Other sex-specific dietary patterns were the “whole grain” pattern for men and the “white rice” pattern for women. Women adhering to the “prudent” pattern had a 25% lower risk of hyperglycemia incidence, whereas those adhering to the “fatty fish, meat, and flour-based food” pattern had a 22% higher risk of developing hyperglycemia. However, these significant associations were not found in men. Other two dietary patterns were not prospectively associated with hyperglycemia risk either in men or women.

In this study, individuals who had the “prudent” pattern consumed more green and yellow vegetables, light-colored vegetables, seaweeds, mushrooms, fruits, legumes and soy products, bone fish, and milk than those who did not adhere to this dietary pattern. The “prudent” pattern derived in this study had components similar to those of the “prudent” or “healthy” pattern, which was characterized by high intake of vegetables, fruits, soy products, and dairy products, from several European [18] and Asian [19,20] studies. Similar to our findings, these prospective studies suggested the inverse relationships between the “prudent” or “healthy” dietary pattern and T2DM incidence. The present results were further supported by literature indicating that the major food components of the “prudent” pattern have been associated with a reduced risk of T2DM, even though a single food and nutrient approach still has potential limitations. Consumption of vegetables and fruits was significantly associated with a decreased incidence of T2DM in various populations [21,22]. The high content of dietary fiber and potassium in vegetables and fruits is beneficial in lowering the future risk of T2DM [23,24]. Seaweed consumption was inversely associated with a risk of T2DM in nationally representative samples of Korean adults [25]. A randomized controlled trial has also reported that T2DM patients using seaweed supplementation had favorably low blood glucose levels relative to a control group [26]. Furthermore, a recent meta-analysis demonstrated that milk and yogurt, the key components of the “prudent” pattern, may have a protective effect on the incidence of T2DM [27].

We found that the “fatty fish, meat, and flour-based food” pattern, characterized mainly by fatty fish, high-fat red meat, processed meats, bread, and noodles/dumplings, was positively associated with the increased risk of hyperglycemia development. These findings were in line with the other observations indicating that the “Western” dietary patterns were associated with T2DM incidence [28,29,30]. The key foods included in the “fatty fish, meat, and flour-based food” pattern were comparable to the major constituents of the “Western” dietary pattern defined in previous studies, and dietary constituents such as fatty fish and red and processed meats may be partially responsible for the negative effects on T2DM development. The health benefits and risks of fish consumption are still being debated [31]. Consumption of fish, as a great source of omega-3 fatty acids, has beneficial effects on lipid profiles and cardiovascular health [32,33]. On the contrary, fatty fish is a major source of exposure to persistent organic pollutants (POPs), which act as potential endocrine disruptors [34]. In previous cross-sectional studies, exposure to POPs is significantly associated with higher risk of insulin resistance [35] and T2DM [36]. This finding implies that increased exposure to POPs due to high consumption of fatty fish may lead to a higher risk of future hyperglycemia and T2DM. Our findings were also consistent, to some extent, with the results of previous epidemiological studies, which reported the detrimental effects of red meat consumption. According to the findings from the Nurses’ Health Study by Fung et al., both red meat (relative risk [RR] for Q5 vs. Q1, 1.22 [1.05–1.41]; *p* for trend = 0.03) and processed meat (RR for Q5 vs. Q1, 1.48 [1.27–1.73], *p* for trend <0.001), which are major contributors to the Western pattern, were found to be positively associated with the T2DM incidence [28]. A recent prospective study using the three large prospective cohorts of adults in the United States reported that all red meats and their subtypes (unprocessed and processed) increased the risk of developing T2DM [6].

We found sex-specific associations between dietary patterns and future hyperglycemia risks. Although the exact biological mechanisms for this remain unclear, sex differences might be partly explained by the following potential reasons. First, there are sex differences in dietary intakes and behaviors [37,38], and these differences may differentially influence the diet-mediated pathology of T2DM. In a previous study conducted in 27,585 Japanese adults, women who consumed soft drinks almost every day had a greater risk of 5- and 10-year incident T2DM than non-consumers, but such association was not reported in men [12]. Another study from the Western population also reported significantly inverse association of fruit and vegetable consumption with T2DM risk only in women, while they did not find any association in men [39]. Second, sex-specific relations between dietary patterns and hyperglycemia risk might be explained by endogenous sex hormones. Lower levels of testosterone in men and higher levels of testosterone in women increased the risk of T2DM incidence [40,41], and estradiol levels were significantly associated with the risk of T2DM in women, but not in men [42,43]. Third, sex differences in gene expressions related to the pathology of T2DM could be responsible for the heterogeneous associations by sex. According to a recent genome-wide association meta-analysis, there was sexual dimorphism in gene expression in tissues related to insulin resistance, which might trigger the development of T2DM [44]. Findings from the present study highlight the importance of considering the differential effects of sex on progression of T2DM. Changing dietary pattern should be considered as high-priority for hyperglycemia and T2DM prevention especially for women in which other modifiable risk factors such as smoking and heavy drinking is not common. For women, changing from a diet that is rich in meat, fatty fish, and bread to one that is plentiful in vegetables, mushrooms, and seaweeds might be beneficial in preventing future T2DM, which is in line with current dietary recommendations [15].

The present study has some limitations. First, the factor analysis conducted in this study to ascertain dietary patterns has a well-known weakness of arbitrary decisions involved at each step such as aggregating food items into food groups, choosing the number of factors to retain and the rotation method, and labelling the derived dietary patterns. To overcome this issue, we aggregated food items into food groups based on their nutritional profiles and culinary uses considered in the previous studies [10,16,17] and determined the number of factors to retain based on the eigenvalue, scree test results, and interpretability of each dietary pattern. We also applied orthogonal rotation (varimax options) to ensures better interpretability and orthogonality of the matrix of factor loadings [30] and labelled the dietary patterns based on food groups which were the most positively loaded on each factor and the name of patterns identified in previous studies [18,29]. Furthermore, the usefulness of dietary pattern analysis in diet-disease association studies has been well documented, and results of previous studies investigating the relationships between dietary patterns and T2DM align with the current knowledge [45,46]. Second, the foods listed on the FFQ were determined in advance and might not fully capture individual variability [47]. However, since the FFQ used in the KoGES study was developed based on the foods frequently consumed by representative samples of Korean adults aged 40–69 years, it may efficiently cover the dietary intakes of our study participants of the same age group. Moreover, the FFQ used in this study was previously validated to reflect a usual dietary intake and was well correlated with various biomarkers and diseases in previous studies [48,49]. Third, we used the dietary data from a single measurement of the FFQ at the baseline examination. Although individual’s dietary behaviors and food intake could change over time, dietary patterns derived at a population level are stable and reproducible over time [50,51].

Despite the limitations mentioned above, this study is the first to prospectively investigate the sex-specific associations between dietary patterns and hyperglycemia risk among Korean adults. Several strengths of the present study included the use of a considerably large population-based cohort and dietary data measured by a validated FFQ, and adjustments for multiple covariates related to dietary intake and hyperglycemia. Although the KoGES-HEXA data used in the current study may not be representative of entire Korean populations, we targeted a number of adults living in urban cities who had health examination at medical centers in all over the country. Furthermore, our analytic models included baseline fasting glucose level, which is a strong predictor for T2DM [52], but have not been taken into account in previous prospective studies investigating dietary patterns associated with T2DM.

## 5. Conclusions

In conclusion, this prospective cohort study identified sex-specific dietary patterns that were significantly associated with a risk of hyperglycemia among middle-aged and older Korean adults. Results from this study indicated that in women, the “prudent” pattern, which included high intake of vegetables, mushrooms, seaweeds, fruits, and soy products, may potentially play a protective role against hyperglycemia development, whereas the “fatty fish, meat, and flour-based food” pattern is associated with an increased incidence of hyperglycemia. Our findings were based on the overall dietary patterns rather than individual foods or nutrients. The findings of this study might provide useful information for guiding food choices and dietary intake to prevent T2DM through nutritional education and intervention, especially for middle-aged and older Korean women.

## Figures and Tables

**Table 1:** Factor loadings for the major dietary patterns identified according to a factor analysis ^a^.

	Men				Women			
	Factor 1	Factor 2	Factor 3	Factor 4	Factor 1	Factor 2	Factor 3	Factor 4
	“Prudent” Pattern	“Fatty Fish, Meat, and Flour-Based Food” Pattern	“Coffee and Sweets” Pattern	“Whole Grain” Pattern	“Prudent” Pattern	“Fatty Fish, Meat, and Flour-Based Food” Pattern	“Coffee and Sweets” Pattern	“White Rice” Pattern
Light-colored vegetables	0.702 ^b^				0.678			
Green/yellow vegetables	0.699				0.732			
Lean fish	0.607				0.539			
Seaweeds	0.599				0.599			
Mushrooms	0.551				0.562			
Shellfish	0.517				0.426			
Kimchi	0.501				0.430			
Bone fish	0.493				0.531			
Pickled vegetables	0.429				0.342			
Fruits	0.412				0.457			
Tubers	0.393				0.451			
Legumes and soy products	0.353				0.479			
Milk	0.323				0.324			
Salt-fermented fish	0.307							
Yogurt					0.333			
Fatty fish		0.583			0.318	0.505		
Pizza/hamburger		0.563				0.482		
Processed meats		0.550				0.533		
High-fat red meat		0.481				0.569		
Bread		0.471				0.486		
Poultry		0.466				0.496		
Red meat by-products		0.460				0.475		
Cake/snack/cookie		0.456				0.475		
Noodles/dumpling		0.405				0.404		
Dairy products		0.404						
Other seafood		0.384				0.418		
Carbonated beverages		0.381				0.331		
Red meat		0.327				0.310		
Sweets			0.896				0.861	
Oils/fats			0.894				0.864	
Coffee			0.848				0.785	
Whole grain				0.858				−0.902
White rice				−0.878				0.916
Variance of intake explained (%)	11.68	7.79	6.42	5.29	11.59	7.60	5.94	4.68
Cumulative variance of intake explained (%)	11.68	19.47	25.88	31.17	11.59	19.19	25.13	29.81

^a^ Factor analysis was performed based on 37 food groups. ^b^ Factor loading values of <|0.300| were not listed in the table for simplicity.

**Table 2:** General characteristics at baseline across quintiles of each dietary pattern score in the middle-aged Korean men, the KoGES-HEXA study.

	Quintile (Q) of Dietary Pattern Score			*p*-Value ^b^
	Q1 (Lowest)	Q3	Q5 (Highest)	
**“Prudent” pattern**				
Age, years	53.4 ± 8.7 ^a^	54.9 ± 8.4	55.4 ± 8.3	<0.0001
Education, college or higher, %	43.9	45.2	47.6	<0.0001
Current smokers, %	27.8	26.1	27.3	0.9834
Alcohol, g/day	13.9 ± 24.4	15.5 ± 24.5	18.7 ± 30.6	<0.0001
Regular physical activity, %	53.7	59.9	65.5	<0.0001
Family history of diabetes, %	14.9	13.6	13.0	0.0227
Body mass index, kg/m^2^	24.2 ± 2.7	24.3 ± 2.7	24.5 ± 2.6	<0.0001
**“Fatty fish, meat, and flour-based food” pattern**				
Age, years	58.7 ± 7.4	54.8 ± 7.9	50.2 ± 8.3	<0.0001
Education, college or higher, %	35.0	43.9	56.0	<0.0001
Current smokers, %	22.3	26.9	32.8	<0.0001
Alcohol, g/day	13.6 ± 25.4	16.8 ± 27.1	18.6 ± 30.5	<0.0001
Regular physical activity, %	59.2	59.3	57.2	0.0261
Family history of diabetes, %	11.3	13.0	16.9	<0.0001
Body mass index, kg/m^2^	24.1 ± 2.6	24.3 ± 2.6	24.6 ± 2.8	<0.0001
**“Coffee and sweets” pattern**				
Age, years	54.9 ± 8.5	54.2 ± 8.5	53.5 ± 8.4	<0.0001
Education, college or higher, %	46.0	50.2	39.7	<0.0001
Current smokers, %	16.0	26.9	43.9	<0.0001
Alcohol, g/day	17.5 ± 33.1	16.8 ± 25.9	16.5 ± 29.6	0.0119
Regular physical activity, %	63.4	60.2	52.8	<0.0001
Family history of diabetes, %	13.3	14.8	13.4	0.5869
Body mass index, kg/m^2^	24.0 ± 2.7	24.5 ± 2.7	24.4 ± 2.6	<0.0001
**“Whole grain” pattern**				
Age, years	53.2 ± 8.6	55.6 ± 8.2	54.8 ± 8.5	<0.0001
Education, college or higher, %	40.5	41.2	52.8	<0.0001
Current smokers, %	33.9	26.1	21.5	<0.0001
Alcohol, g/day	20.0 ± 34.2	16.8 ± 27.9	12.3 ± 22.0	<0.0001
Regular physical activity, %	47.9	60.1	67.5	<0.0001
Family history of diabetes, %	13.2	12.4	15.0	0.1031
Body mass index, kg/m^2^	24.3 ± 2.6	24.3 ± 2.6	24.4 ± 2.6	0.1125

KoGES, Korean Genome and Epidemiology Study; HEXA, Health Examinee. ^a^ Mean ± standard deviation (all such values). ^b^ *p*-values were obtained from the multiple linear regression for continuous variables and the Mantel–Haenszel chi-square test for categorical variables.

**Table 3:** General characteristics at baseline across quintiles of each dietary pattern score in the middle-aged Korean women, the KoGES-HEXA study.

	Quintile (Q) of Dietary Pattern Score			*p*-Value ^b^
	Q1 (Lowest)	Q3	Q5 (Highest)	
**“Prudent” pattern**				
Age, years	51.6 ± 7.9 ^a^	52.7 ± 7.6	52.9 ± 7.3	<0.0001
Education, college or higher, %	24.3	24.5	27.2	<0.0001
Current smokers, %	2.2	1.6	1.5	0.0016
Alcohol, g/day	1.8 ± 6.7	1.7 ± 7.3	1.6 ± 6.1	0.1220
Regular physical activity, %	44.7	53.5	62.4	<0.0001
Family history of diabetes, %	18.9	18.3	19	0.7505
Body mass index, kg/m^2^	23.3 ± 2.9	23.5 ± 2.8	23.6 ± 2.8	<0.0001
**“Fatty fish, meat, and flour-based food” pattern**				
Age, years	56.0 ± 7.0	52.5 ± 7.3	48.7 ± 7.0	<0.0001
Education, college or higher, %	15.7	24.1	36.9	<0.0001
Current smokers, %	1.1	1.6	2.3	<0.0001
Alcohol, g/day	0.9 ± 4.7	1.7 ± 6.2	2.6 ± 7.7	<0.0001
Regular physical activity, %	55.7	53.7	50.1	<0.0001
Family history of diabetes, %	16.2	19.7	22.3	<0.0001
Body mass index, kg/m^2^	23.7 ± 2.8	23.4 ± 2.8	23.3 ± 3.0	<0.0001
**“Coffee and sweets” pattern**				
Age, years	53.9 ± 7.6	51.8 ± 7.3	52.0 ± 7.7	<0.0001
Education, college or higher, %	20.3	28.2	24.8	<0.0001
Current smokers, %	1.0	1.5	2.8	<0.0001
Alcohol, g/day	1.4 ± 6.6	1.9 ± 5.8	1.9 ± 8.1	<0.0001
Regular physical activity, %	54.2	56.1	48.1	<0.0001
Family history of diabetes, %	18.2	20.2	18.4	0.8811
Body mass index, kg/m^2^	23.2 ± 2.8	23.6 ± 2.8	23.7 ± 3.0	<0.0001
**“White rice” pattern**				
Age, years	51.4 ± 7.7	53.0 ± 7.6	51.8 ± 7.6	<0.0001
Education, college or higher, %	30.2	25.4	23.8	<0.0001
Current smokers, %	1.2	1.6	2.8	<0.0001
Alcohol, g/day	1.4 ± 5.5	1.7 ± 5.8	2.3 ± 8.2	<0.0001
Regular physical activity, %	55.2	54.8	44.6	<0.0001
Family history of diabetes, %	21.0	18.9	17.9	0.0286
Body mass index, kg/m^2^	23.4 ± 2.8	23.5 ± 2.9	23.5 ± 2.9	<0.0001

KoGES, Korean Genome and Epidemiology Study; HEXA, Health Examinee. ^a^ Mean ± standard deviation (all such values). ^b^ *p*-values were obtained from the multiple linear regression for continuous variables and the Mantel–Haenszel chi-square test for categorical variables.

**Table 4:** Energy and nutrient intake at baseline across quintiles of each dietary pattern score in the middle-aged Korean men, the KoGES-HEXA study.

	Quintile (Q) of Dietary Pattern Score			*p*-Value ^b^
	Q1 (Lowest)	Q3	Q5 (Highest)	
**“Prudent” pattern**				
Total energy, kcal	1593 ± 457 ^a^	1801 ± 415	2254 ± 580	<0.0001
Carbohydrate, % of energy	74.7 ± 5.9	71.7 ± 5.8	67.1 ± 7.1	<0.0001
Protein, % of energy	11.3 ± 1.6	13.1 ± 1.7	15.8 ± 2.6	<0.0001
Fat, % of energy	5.3 ± 2.3	6.2 ± 2.1	7.5 ± 2.3	<0.0001
Calcium, mg	278.4 ± 127.8	399.7 ± 141.3	537.3 ± 201.8	<0.0001
Phosphorus, mg	742.3 ± 109.5	871.0 ± 116.1	1028.2 ± 166.3	<0.0001
Iron, mg	7.4 ± 1.7	9.4 ± 1.9	12.3 ± 3.6	<0.0001
Sodium, mg	1781 ± 977	2533 ± 1065	3302 ± 1412	<0.0001
Potassium, mg	1562 ± 478	2129 ± 484	2765 ± 670	<0.0001
Vitamin A, RAE	273.2 ± 141.9	444.9 ± 213.7	683.6 ± 361.3	<0.0001
Carotene, µg	1272 ± 734	2212 ± 1199	3564 ± 2107	<0.0001
Vitamin C, mg	55.9 ± 31.1	93.7 ± 37.9	134.2 ± 55.3	<0.0001
Folate, g	138.9 ± 52.3	201.4 ± 65.1	279.4 ± 102.6	<0.0001
Dietary fiber, g	3.9 ± 1.3	5.4 ± 1.6	7.2 ± 2.4	<0.0001
**“Fatty fish, meat, and flour-based food” pattern**				
Total energy, kcal	1612 ± 395	1763 ± 385	2329 ± 596	<0.0001
Carbohydrate, % of energy	75.9 ± 5.2	71.9 ± 5.3	65.6 ± 6.6	<0.0001
Protein, % of energy	12.7 ± 2.5	13.1 ± 2.2	14.5 ± 2.5	<0.0001
Fat, % of energy	4.5 ± 1.7	6.1 ± 1.8	8.5 ± 2.2	<0.0001
Calcium, mg	453.4 ± 226.0	383.9 ± 165.3	389.0 ± 136.9	<0.0001
Phosphorus, mg	906.3 ± 187.3	859.8 ± 150.9	881.6 ± 139.9	<0.0001
Iron, mg	10.6 ± 3.9	9.2 ± 2.4	9.5 ± 2.3	<0.0001
Sodium, mg	3160 ± 1626	2355 ± 1074	2328 ± 896	<0.0001
Potassium, mg	2424 ± 832	2059 ± 609	2042 ± 514	<0.0001
Vitamin A, RAE	566.3 ± 404.3	422.4 ± 226.9	430.3 ± 188.5	<0.0001
Carotene, µg	3057 ± 2295	2092 ± 1244	2007 ± 1053	<0.0001
Vitamin C, mg	118.1 ± 62.4	88.9 ± 42.6	83.1 ± 37.0	<0.0001
Folate, g	246.2 ± 117.6	192.7 ± 72.6	190.9 ± 63.3	<0.0001
Dietary fiber, g	6.8 ± 2.6	5.2 ± 1.7	4.9 ± 1.5	<0.0001
**“Coffee and sweets” pattern**				
Total energy, kcal	1743 ± 531	1879 ± 509	1995 ± 530	<0.0001
Carbohydrate, % of energy	71.8 ± 7.3	70.8 ± 6.6	70.6 ± 6.4	<0.0001
Protein, % of energy	13.5 ± 2.8	13.6 ± 2.4	13.0 ± 2.3	<0.0001
Fat, % of energy	5.9 ± 2.5	6.4 ± 2.3	6.8 ± 2.2	<0.0001
Calcium, mg	400.6 ± 193.6	410.7 ± 176.6	390.9 ± 156.6	<0.0001
Phosphorus, mg	873.7 ± 175.6	885.5 ± 155.7	873.4 ± 144.6	0.0014
Iron, mg	9.7 ± 3.1	9.7 ± 2.9	9.4 ± 2.7	<0.0001
Sodium, mg	2392 ± 1219	2555 ± 1216	2573 ± 1166	<0.0001
Potassium, mg	1998 ± 720	2197 ± 658	2241 ± 612	<0.0001
Vitamin A, RAE	457.8 ± 274.1	463.2 ± 281.0	442.6 ± 257.4	0.0002
Carotene, µg	2267 ± 1515	2309 ± 1586	2228 ± 1482	0.0044
Vitamin C, mg	94.4 ± 52.3	96.2 ± 49.4	90.6 ± 45.5	<0.0001
Folate, g	207.2 ± 90.0	206.8 ± 87.1	195.9 ± 78.0	<0.0001
Dietary fiber, g	5.5 ± 2.1	5.5 ± 2.0	5.3 ± 1.9	<0.0001
**“Whole grain” pattern**				
Total energy, kcal	1754 ± 490	1700 ± 396	2261 ± 487	<0.0001
Carbohydrate, % of energy	71.0 ± 7.3	73.1 ± 6.1	71.8 ± 5.6	<0.0001
Protein, % of energy	13.2 ± 2.7	12.9 ± 2.2	12.9 ± 1.9	<0.0001
Fat, % of energy	6.3 ± 2.5	5.6 ± 2.1	6.4 ± 2.0	<0.0001
Calcium, mg	358.5 ± 156.0	366.4 ± 171.3	427.4 ± 169.1	<0.0001
Phosphorus, mg	856.0 ± 151.9	848.3 ± 149.4	867.7 ± 146.8	<0.0001
Iron, mg	8.6 ± 2.7	9.4 ± 2.7	9.4 ± 2.5	<0.0001
Sodium, mg	2634 ± 1302	2468 ± 1157	2152 ± 860	<0.0001
Potassium, mg	2006 ± 619	2040 ± 646	2126 ± 595	<0.0001
Vitamin A, RAE	443.4 ± 277.3	429.0 ± 256.3	417.2 ± 205.0	<0.0001
Carotene, µg	2230 ± 1579	2202 ± 1454	2033 ± 1180	<0.0001
Vitamin C, mg	87.6 ± 44.5	87.9 ± 45.7	94.3 ± 48.0	<0.0001
Folate, g	192.5 ± 84.7	195.3 ± 80.0	199.4 ± 70.9	<0.0001
Dietary fiber, g	5.0 ± 2.0	5.4 ± 1.9	5.4 ± 1.8	<0.0001

KoGES, Korean Genome and Epidemiology Study; HEXA, Health Examinee. ^a^ Mean ± standard deviation (all such values). ^b^ *p*-values were obtained from the multiple linear regression.

**Table 5:** Energy and nutrient intake at baseline across quintiles of each dietary pattern score in the middle-aged Korean women, the KoGES-HEXA study.

	Quintile (Q) of Dietary Pattern Score			*p*-Value ^b^
	Q1 (Lowest)	Q3	Q5 (Highest)	
**“Prudent” pattern**				
Total energy, kcal	1402 ± 454 ^a^	1654 ± 414	2137 ± 576	<0.0001
Carbohydrate, % of energy	75.0 ± 6.6	72.3 ± 6.1	68.5 ± 7.4	<0.0001
Protein, % of energy	11.6 ± 1.9	13.3 ± 2.0	15.7 ± 2.8	<0.0001
Fat, % of energy	5.2 ± 2.5	6.0 ± 2.2	7.1 ± 2.4	<0.0001
Calcium, mg	282.7 ± 121.2	432.1 ± 148.5	601.8 ± 206.4	<0.0001
Phosphorus, mg	699.3 ± 107.2	834.2 ± 122.8	997.0 ± 175.9	<0.0001
Iron, mg	7.2 ± 1.7	9.3 ± 2.0	12.2 ± 3.4	<0.0001
Sodium, mg	1671 ± 926	2303 ± 1022	2950 ± 1264	<0.0001
Potassium, mg	1525 ± 452	2135 ± 508	2847 ± 685	<0.0001
Vitamin A, RAE	273.2 ± 147.2	4339 ± 200.7	662.3 ± 325.6	<0.0001
Carotene, µg	1302 ± 800	2149 ± 1138	3427 ± 1908	<0.0001
Vitamin C, mg	64.5 ± 35.0	105.9 ± 44.9	151.5 ± 61.0	<0.0001
Folate, g	141.5 ± 52.7	205.5 ± 64.7	287.4 ± 97.1	<0.0001
Dietary fiber, g	4.0 ± 1.3	5.5 ± 1.6	7.4 ± 2.3	<0.0001
**“Fatty fish, meat, and flour-based food” pattern**				
Total energy, kcal	1525 ± 446	1589 ± 409	2160 ± 602	<0.0001
Carbohydrate, % of energy	76.3 ± 5.6	72.8 ± 5.4	65.8 ± 6.8	<0.0001
Protein, % of energy	12.8 ± 2.5	13.2 ± 2.3	14.8 ± 2.7	<0.0001
Fat, % of energy	4.5 ± 1.8	5.8 ± 1.8	8.4 ± 2.3	<0.0001
Calcium, mg	520.5 ± 240.2	416.4 ± 178.5	409.4 ± 147.7	<0.0001
Phosphorus, mg	881.7 ± 198.1	823.5 ± 159.1	850.9 ± 148.6	<0.0001
Iron, mg	10.3 ± 3.7	9.1 ± 2.6	9.5 ± 2.4	<0.0001
Sodium, mg	2765 ± 1404	2177 ± 1060	2175 ± 861	<0.0001
Potassium, mg	2491 ± 837	2077 ± 656	2069 ± 550	<0.0001
Vitamin A, RAE	540.0 ± 345.7	422.8 ± 233.0	434.2 ± 202.7	<0.0001
Carotene, µg	2864 ± 1990	2092 ± 1292	2050 ± 1127	<0.0001
Vitamin C, mg	133.5 ± 68.7	101.7 ± 51.0	95.1 ± 42.7	<0.0001
Folate, g	248.1 ± 108.9	199.1 ± 78.1	195.8 ± 66.2	<0.0001
Dietary fiber, g	6.8 ± 2.5	5.3 ± 1.8	5.0 ± 1.6	<0.0001
**“Coffee and sweets” pattern**				
Total energy, kcal	1614 ± 548	1694 ± 511	1812 ± 531	<0.0001
Carbohydrate, % of energy	72.1 ± 8.0	71.8 ± 6.7	72.0 ± 6.4	<0.0001
Protein, % of energy	13.8 ± 3.1	13.6 ± 2.5	12.9 ± 2.3	<0.0001
Fat, % of energy	5.8 ± 2.7	6.1 ± 2.3	6.4 ± 2.3	<0.0001
Calcium, mg	412.8 ± 191.6	453.6 ± 194.9	429.5 ± 174.5	<0.0001
Phosphorus, mg	833.0 ± 181.6	853.5 ± 165.5	830.8 ± 151.7	<0.0001
Iron, mg	9.5 ± 3.1	9.7 ± 2.9	9.1 ± 2.5	<0.0001
Sodium, mg	2109 ± 1077	2351 ± 1113	2357 ± 1090	<0.0001
Potassium, mg	1963 ± 695	2248 ± 707	2232 ± 641	<0.0001
Vitamin A, RAE	444.5 ± 269.2	460.3 ± 259.1	430.6 ± 239.7	<0.0001
Carotene, µg	2221 ± 1496	2293 ± 1473	2152 ± 1376	<0.0001
Vitamin C, mg	101.7 ± 53.6	111.3 ± 57.0	103.3 ± 50.8	<0.0001
Folate, g	205.5 ± 87.1	215.1 ± 86.5	200.8 ± 78.2	<0.0001
Dietary fiber, g	5.5 ± 2.0	5.7 ± 2.1	5.4 ± 1.9	<0.0001
**“White rice” pattern**				
Total energy, kcal	2074 ± 472	1752 ± 437	1574 ± 584	<0.0001
Carbohydrate, % of energy	73.5 ± 5.6	71.8 ± 6.0	69.9 ± 8.4	<0.0001
Protein, % of energy	12.3 ± 1.8	13.7 ± 2.1	14.2 ± 3.4	<0.0001
Fat, % of energy	5.8 ± 2.0	6.1 ± 2.2	6.8 ± 2.8	<0.0001
Calcium, mg	364.9 ± 133.9	442.5 ± 156.8	476.3 ± 239.8	<0.0001
Phosphorus, mg	765.0 ± 120.4	852.4 ± 130.5	885.8 ± 211.9	<0.0001
Iron, mg	8.2 ± 1.8	9.8 ± 2.3	9.8 ± 4.0	<0.0001
Sodium, mg	1827 ± 781	2348 ± 1023	2613 ± 1369	<0.0001
Potassium, mg	1832 ± 478	2196 ± 535	2324 ± 879	<0.0001
Vitamin A, RAE	334.6 ± 153.5	464.8 ± 219.8	524.2 ± 345.1	<0.0001
Carotene, µg	1614 ± 872	2351 ± 1306	2618 ± 1943	<0.0001
Vitamin C, mg	83.6 ± 38.1	108.0 ± 45.9	119.6 ± 67.7	<0.0001
Folate, g	171.9 ± 57.4	213.4 ± 71.6	228.4 ± 110.5	<0.0001
Dietary fiber, g	4.7 ± 1.4	5.7 ± 1.8	5.8 ± 2.6	<0.0001

KoGES, Korean Genome and Epidemiology Study; HEXA, Health Examinee. ^a^ Mean ± standard deviation (all such values). ^b^ *p*-values were obtained from the multiple linear regression.

**Table 6:** Adjusted hazard ratios (95% confidence intervals) of hyperglycemia across quintiles of each dietary pattern score in the middle-aged Korean adults, the KoGES-HEXA study.

	Men				Women			
	Quintile (Q) of Dietary Pattern Score			*p* for Trend	Quintile (Q) of Dietary Pattern Score			*p* for Trend
	Q1 (Lowest)	Q3	Q5 (Highest)		Q1 (Lowest)	Q3	Q5 (Highest)	
“Prudent” pattern								
Person-years	18,246	17,750	18,001	-	36,520	36,346	38,230	-
Hyperglycemia (cases)	231	240	222	-	282	271	282	-
Age-adjusted HR (95% CI) ^a^	1.00	1.10 (0.92–1.32)	1.00 (0.83–1.20)	0.9650	1.00	0.98 (0.83–1.15)	0.85 (0.72–1.01)	0.0427
Multivariate-adjusted HR (95% CI)	1.00	1.07 (0.89–1.29)	0.93 (0.75–1.15)	0.4457	1.00	0.91 (0.77–0.99)	0.75 (0.63–0.89)	0.0003
“Fatty fish, meat, and flour-based food” pattern								
Person-years	18,229	17,868	17,658	-	37,780	36,471	36,906	-
Hyperglycemia (cases)	245	248	210	-	305	274	265	-
Age-adjusted HR (95% CI)	1.00	1.15 (0.96–1.37)	1.06 (0.88–1.29)	0.5544	1.00	1.27 (1.08–1.50)	1.39 (1.17–1.65)	0.0002
Multivariate-adjusted HR (95% CI)	1.00	1.10 (0.91–1.32)	1.04 (0.83–1.30)	0.6834	1.00	1.13 (0.92–1.38)	1.22 (1.03–1.44)	0.0210
“Coffee and sweets” pattern								
Person-years	18,161	18,187	17,430	-	37,000	36,453	36,618	-
Hyperglycemia (cases)	210	252	218	-	304	270	290	-
Age-adjusted HR (95% CI)	1.00	1.21 (1.01–1.46)	1.19 (0.99–1.44)	0.1259	1.00	1.06 (0.90–1.25)	1.09 (0.93–1.28)	0.0177
Multivariate-adjusted HR (95% CI)	1.00	1.20 (0.99–1.45)	1.06 (0.87–1.30)	0.7622	1.00	0.97 (0.82–1.15)	0.94 (0.80–1.11)	0.7350
“Whole grain (men)/white rice (women)” pattern								
Person-years	18,411	17,986	17,442	-	35,949	36,887	37,756	-
Hyperglycemia (cases)	247	241	200	-	245	298	308	-
Age-adjusted HR (95% CI)	1.00	1.06 (0.89–1.26)	0.89 (0.74–1.08)	0.3313	1.00	1.03 (0.87–1.22)	1.04 (0.88–1.23)	0.3762
Multivariate-adjusted HR (95% CI)	1.00	0.99 (0.82–1.19)	0.98 (0.80–1.21)	0.9672	1.00	1.01 (0.84–1.20)	0.99 (0.82–1.19)	0.4459

KoGES, Korean Genome and Epidemiology Study; HEXA, Health Examinee; HR, hazard ratio; CI, confidence interval. ^a^ Age-adjusted model was adjusted for age (years); multivariable-adjusted model was additionally adjusted for education (≤elementary school; middle school; high school; ≥college), smoking habits (never, past, or current), total alcohol intake (g/day), regular physical activity (yes or no), family history of diabetes (yes or no), study site, body mass index (kg/m^2^), fasting blood glucose levels (mg/dL), and total energy intake (kcal/day) at baseline.

## Supplementary Materials

The following are available online at https://www.mdpi.com/2072-6643/11/8/1801/s1, Table S1: Distribution of factor scores across quintiles of each dietary pattern in the middle-aged Korean adults, the KoGES-HEXA study.
