# Supplementary material for: Identification of Dietary Patterns Associated with Incidence of Hyperglycemia in Middle-Aged and Older Korean Adults
Source: Nutrients. 2019 Aug 4;11(8):1801. doi: 10.3390/nu11081801 (PMC6723075; doi:10.3390/nu11081801)
Supplement: Supplementary file 1 [file nutrients-11-01801-s001.pdf]

Identification of Dietary Patterns Associated with Incidence of Type 2 Diabetes in Middle-Aged and Older Korean Adults

Authors: Kyung Won Lee, PhD, Hae Dong Woo, PhD, Mi Jin Cho, MS, Jae Kyung Park, MS, Sung Soo Kim, PhD*

Supplementary Table S1. Distribution of factor scores across quintiles of each dietary pattern in the middle-aged Korean adults, the KoGES-HEXA study

	Men			Women		
	Quintile (Q) of dietary pattern score			Quintile (Q) of dietary pattern score		
	Q1 (lowest)	Q3	Q5 (highest)	Q1 (lowest)	Q3	Q5 (highest)
"Prudent" pattern						
Median	-0.91	-0.20	1.11	-0.93	-0.20	1.09
Range	-3.24 to -0.71	-0.37 to -0.01	0.54 to 21.16	-5.60 to -0.71	-0.37 to 0.00	0.55 to 19.32
"Fatty fish, meat, and flour-based food" pattern						
Median	-0.80	-0.23	1.04	-0.74	-0.23	0.95
Range	-3.66 to -0.64	-0.37 to -0.06	0.45 to 27.88	-3.53 to -0.59	-0.36 to -0.07	0.42 to 24.57
"Coffee and sweets" pattern						
Median	-1.16	-0.14	1.68	-1.03	-0.17	1.17
Range	-4.55 to -1.01	-0.39 to 0.22	0.74 to 6.25	-7.78 to -0.91	-0.48 to 0.12	0.78 to 8.46
"Whole grain (men)/white rice (women)" pattern						
Median	-1.63	0.31	0.97	-0.75	-0.36	1.91
Range	-8.49 to -1.17	0.18 to 0.42	0.71 to 5.52	-9.36 to -0.64	-0.50 to -0.16	0.29 to 4.60
